# Construction of miRNA-mRNA-TF Regulatory Network for Diagnosis of Gastric Cancer

**DOI:** 10.1155/2021/9121478

**Published:** 2021-11-18

**Authors:** Zhenjie Fu, Yuqin Xu, Yan Chen, Hang Lv, Guiping Chen, Yitao Chen

**Affiliations:** ^1^College of Life Science, Zhejiang Chinese Medical University, Hangzhou 310053, China; ^2^Department of Gastrointestinal Surgery, The First Affiliated Hospital of Zhejiang Chinese Medical University, Hangzhou, Zhejiang, China

## Abstract

Gastric cancer (GC), as an epidemic cancer worldwide, has more than 1 million new cases and an estimated 769,000 deaths worldwide in 2020, ranking fifth and fourth in global morbidity and mortality. In mammals, both miRNAs and transcription factors (TFs) play a partial role in gene expression regulation. The mRNA expression profile and miRNA expression profile of GEO database were screened by GEO2R for differentially expressed genes (DEGs) and differentially expressed miRNAs (DEMs). Then, DAVID annotated the functions of DEGs to understand the functions played in biological processes. The prediction of potential target genes of miRNA and key TFs of mRNA was performed by mipathDB V2.0 and CHEA3, respectively, and the gene list comparison was performed to look for overlapping genes coregulated by key TFs and DEMs. Finally, the obtained miRNAs, TF, and overlapping genes were used to construct the miRNA-mRNA-TF regulatory network, which was verified by RT-qPCR. 76 upregulated DEGs, 199 downregulated DEGs, and 3 upregulated miRNAs (miR-199a-3p/miR-199b-3p, miR-125b-5p, and miR-199a-5p) were identified from the expression profiles of mRNA (GSE26899, GSE29998, GSE51575, and GSE13911) and miRNA (GSE93415), respectively. Through database prediction and gene list comparison, it was found that among the 199 downregulated DEGs, 61, 71, and 69 genes were the potential targets of miR-199a-3p/miR-199b-3p, miR-125b-5p, and miR-199a-5p, respectively. 199 downregulated DEGs were used as the gene list for the prediction of key TFs, and the results showed that RFX6 ranked the highest. The potential target overlap genes of miR-199a-3p/miR-199b-3p, miR-125b-5p, and miR-199a-5p were 4 genes (SH3GL2, ATP4B, CTSE, and SORBS2), 7 genes (SLC7A8, RNASE4, ESRRG, PGC, MUC6, Fam3B, and FMO5), and 6 genes (CHGA, PDK4, TMPRSS2, CLIC6, GPX3, and PSCA), respectively. Finally, we constructed a miRNA-mRNA-TF regulatory network based on the above 17 mRNAs, 3 miRNAs, and 1 TF and verified by RT-qPCR and western blot results that the expression of RFX6 was downregulated in GC tissues. These identified miRNAs, mRNAs, and TF have a certain reference value for further exploration of the regulatory mechanism of GC.

## 1. Introduction

Gastric cancer (GC), as an epidemic cancer worldwide, has more than 1 million new cases and an estimated 769,000 deaths worldwide in 2020, ranking fifth and fourth in global morbidity and mortality [1]. Despite great advances in our understanding of cancer, the causes of its occurrence and progression are not fully understood. In general, the related factors involved in the occurrence and development of GC can be divided into genetic and epigenetic changes, as well as environmental and lifestyle factors [2, 3]. The molecular pathogenesis of GC has always been the focus of scientists. In recent years, miRNA and transcription factors (TFs) have become an important breakthrough direction in the diagnosis, prognosis, and targeted therapy of GC.

As a class of small noncoding RNAs (ncRNAs) composed of 17-25 nucleotides [4], miRNAs regulate mRNA and protein levels by guiding Argonaute (AGO) proteins to the 3′-UTR region of mRNA leading to mRNA degradation [4–7]. Since the incidental discovery of miRNAs in *Caenorhabditis elegans* [8–12], accumulated evidence has shown that miRNAs play a key regulatory role in biological processes in animals, especially in recent years, new achievements have been made in the study of miRNAs and human cancers [13, 14]. Previous studies have shown that in the diagnosis of GC, miR-940 showed 81.25% sensitivity and 98.57% specificity. The AUC, sensitivity, and specificity of miR-21, miR-93, miR-106a, and miR-106b combinations were 0.887, 84.80%, and 79.2%, respectively [15, 16]. In addition, the expression of miR-204 (HR = 3.900, 95% CI 1.300-11.800) and miR-15a (RR = 1.950, 95% CI 0.470 9.130) was strongly associated with poor survival in patients with GC [17, 18]. Therefore, it is very promising to complete the personalized management, diagnosis, and prognosis of GC through miRNA combination in the future [19, 20].

TFs are proteins that bind to chromatin to activate or inhibit transcription by helical binding of DNA to specific regulatory sequences in the form of trans-activated or trans-repressive domains. They are expressed in tissues spatially, temporally, and sequentially during development, cell renewal, or differentiation, and any change in their expression may lead to uncontrolled cell integrity or dynamic homeostasis, leading to pathological changes [21], such as diabetes [22] and cancers [23]. TFs are classified into different families, reflecting the homology of their DNA binding domains, thus, reflecting the binding sequence of DNA [24, 25]. However, in-depth studies have found that TFs can play a double-edged sword role, so they may play both anticancer and oncogenic roles, even though TFs of the same family may have two-way effects on the same cancer [26, 27]. Taking the role of SOX family TFs in the pathological process of GC as an example, since the first discovery of SRY protein as a TF involved in mammalian male determination, up to 20 members of SOX family have been identified in mammals [28, 29]. However, although they share a DNA binding domain consisting of 79 amino acids, their effects on GC cells are quite different [30]. For example, SOX1 and SOX6 have anticancer effects; SOX3, SOX4, and SOX5 have procancer effects; and SOX2, SOX7, and SOX10 have both anticancer and procancer effects [31]. Therefore, to accurately understand the role of a TF in a certain cancer is the focus of cancer mechanism research and treatment in recent years.

The aim of this study is to further understand the direct regulatory relationship between mRNA, miRNA, and TFs. Through the analysis of existing mRNA and miRNA expression profile data in the GEO database, the functional annotation, and TF prediction of DEGs, target gene prediction of DEMs was performed with the help of databases such as DAVID, mipathDB V2.0, and CHEA3, respectively, and the regulation network of miRNA-mRNAs and TF was finally constructed based on the results above.

## 2. Material and Methods

### 2.1. Microarray Data

To identify DEGs and DEMs in the pathogenesis of GC and build a regulatory network of TF-miRNA-mRNA, we searched the Gene Expression Omnibus (GEO) database with keywords such as “gastric cancer,” “mRNA,” and “miRNA.” Only the data sets containing both GC tissue samples and control group information and without any drug intervention before sequencing could meet the criteria. Finally, 4 mRNA data sets (GSE26899, GSE29998, GSE51575, and GSE13911) and 1 miRNA data set (GSE93415) were selected for the follow-up analysis of this work.

### 2.2. Identification of DEGs and DEMs

GEO2R (https://www.ncbi.nlm.nih.gov/geo/geo2r/) is a software based on GEO database to perform differential analysis on expression profile chips. After the processing of 5 data sets using GEO2R, DEGs and DEMs were screened out using adjust *p* value < 0.05, ∣logFC | >1 and adjust *p* value < 0.01, ∣logFC | >1.9 as the criteria, respectively. The *p* value adjusted by Benjamini Hochberg false discovery rate (FDR) method can significantly reduce the false positive rate [32].

### 2.3. Biological Significance Analysis of DEGs

In order to further understand the biological function of DEGs, Gene Ontology (GO) and Kyoto Encyclopedia of Genes and Genomes (KEGG) pathway enrichment analyses were performed via a database for annotation, visualization, and integrated discovery (DAVID, https://david.ncifcrf.gov/home.jsp). The cut-off criterion, *p* value < 0.05, was set to selected GO terms and KEGG pathway enrichment analysis, and then the analysis results were visualized through OriginPro (2021b_Beta7) software and bioinformatics, an online data visualization tool (http://www.bioinformatics.com.cn).

### 2.4. Prediction of miRNA Target Genes

miRpathDB v2.0 (https://mpd.bioinf.uni-sb.de/overview.html) is a multifunctional online miRNA research site with a large database of miRNA target genes. In order to comprehensively and accurately excavate the regulatory relationship between miRNA and mRNA in GC tissues, the potential targets of candidate miRNA were comprehensively predicted by mipathDB v2.0, and the possible miRNA-mRNA regulation was found by comparing potential targets with DEGs.

### 2.5. Prediction of mRNA Transcription Factors

In the process of gene transcription and expression, miRNA and TF, respectively, play a partial regulatory role. Therefore, further digging out key TFs after clarifying the regulatory relationship between miRNA and mRNA is more conducive to in-depth understanding of the pathological mechanism of GC. TF prediction was performed via ChIP-X Enrichment Analysis 3 (ChEA3). ChEA3, whose database contains a collection of gene set libraries generated from multiple sources including TF-gene coexpression from RNAseq studies, TF-target associations from ChIP-seq experiments, and TF-gene cooccurrence computed from crowd-submitted gene lists, is a TFs enrichment analysis tool that ranks TFs associated with user-submitted gene sets.

## 3. Patients and Samples

To further verify the reliability of the above bioinformatics analysis, 4 pairs of clinical tissue samples from GC patients were obtained from the First Affiliated Hospital of Zhejiang Chinese Medical University. After surgical removal from the patient, all tissue samples were stored in a -80°C cryogenic refrigerator and kept cryogenic during the samples transfer and nonexperimental disposal period. All biometric tests of tissue samples were performed with the informed consent of the patients. The Ethics Committee of the First Affiliated Hospital of Zhejiang Chinese Medical University approved the research (approval no.: 2017-K-002-01).

### 3.1. RNA Isolation and Quantitative Reverse Transcription-PCR (RT-qPCR)

Total RNA was extracted from tissue samples by Trizol method. First of all, 0.05 g tissue samples were weighed and put into 1.0 ml Trizol (Carlsbad, California, USA) solution, fully ground, and then stood for 10 min. Then, 200 *μ*l trichloromethane (Xilong Chemical Co., Ltd., Guangdong Province, China) was added, shaken, and stood for 2 min, and the supernatant was extracted by centrifugation at 4°C 12000 rpm for 15 min. Next, add the same amount of isopropyl alcohol (Huadong Medicine Co., Ltd., Zhejiang Province, China) and rest overnight in the -80°C cryogenic refrigerator. The next day, the samples were taken out, and the supernatant was centrifuged at 4°C 12000 rpm for 10 min and washed twice with an appropriate amount of 75% ethanol (Hangzhou Longshan Fine Chemical Co., Ltd., Zhejiang Province, China). Finally, 20-30 *μ*l nuclease-free water (Promega Corporation, Wisconsin, USA) was added for mixing.

Then, Revert Aid First Strand cDNA Synthesis Kit (Waltham, Massachusetts, USA) was used to obtain cDNA by the instructions, and the iTaqTM Universal SYBR@ Green Master Mix kit was used to detect the expression level of mRNA. All primers (RFX6 forward: CATGGCAAGCCGAGGAAGTGTC; RFX6 reverse: GGTATGTGGAGCAGTGTGATGGAG) were synthesized by Sangon Bioengineering (Shanghai) Co., Ltd. GAPDH (GAPDH forward: CGAGCCACATCGCTCAGACA, GAPDH; GAPDH reverse: CTGGTGAAGACGCCAGTGGA) was used as endogenous control, and all results were calculated and expressed as 2^-*ΔΔ*Ct^.

### 3.2. Western Blot

Clinical tissue samples were lysed in RIPA lysis buffer, and protein concentrations were determined using a BCA Protein Assay Kit (Thermo Scientific, MA, USA). Tissue lysates were separated with SDS-PAGE gels and transferred to a polyvinylidene difluoride (PVDF) membrane (Millipore, Eschborn, Germany). The blots were blocked in 5% milk for 1 h at room temperature. PVDF membranes were incubated with the primary antibodies overnight in a cold room at 4°C. Subsequently, bound primary antibodies were reacted with corresponding secondary antibodies for 1 h at room temperature and evaluated with by chemiluminescence.

### 3.3. Statistical Analysis

Statistical analysis was performed through GraphPad Prism (version 6, San Diego, CA) software. Student's *t*-tests were utilized for the comparison of two sample groups. Differences were considered as statistically significant when *p* < 0.05.

## 4. Results

### 4.1. Differentially Expressed Genes

**Figure 1:**
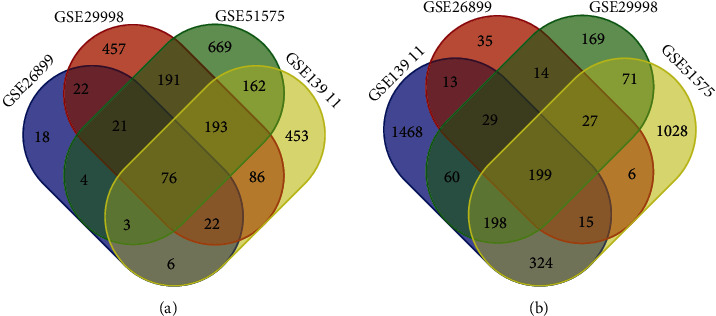
Screening of differentially expressed genes (DEGs). After the data sets of GSE26899, GSE29998, GSE51575, and GSE13911 were processed by GEO2R, the common upregulated DEGs (a) and downregulated DEGs (b) were screened out according to the criteria of adjust *p* value < 0.05 and logFC > 1 and adjust *p* value < 0.05 and logFC < −1, respectively.

Four mRNA expression profiles from GEO database included 210 gastric cancer tissues and 118 normal tissue samples. Four expression profiles (GEO accession nos. GSE26899, GSE29998, GSE51575, and GSE13911) identified 172 upregulated genes and 338 downregulated genes, 1068 upregulated genes and 767 downregulated genes, 1319 upregulated genes and 1868 downregulated genes, and 1001 upregulated genes and 2306 downregulated genes, respectively. Common DEGs were composed of upregulated genes and downregulated genes that overlapped, respectively, among the four data sets. In the end, a total of 275 DEGs were obtained, including 76 upregulated DEGs and 199 downregulated DEGs (Figure 1 and Table 1). In addition, in the miRNA expression profile (GSE93415), three DEMs were screened, which were miR-199a-3p/miR-199b-3p with adjust *p* value = 3.02*E*-04 (log FC = 2.15675), miR-125b-5p with adjust *p* value = 1.58*E*-04 (log FC = 1.98925), and miR-199a-5p adjust *p* value = 9.38*E*-06 (log FC = 1.9549).

### 4.2. GO Terms and KEGG Pathway Enrichment

**Figure 2:**
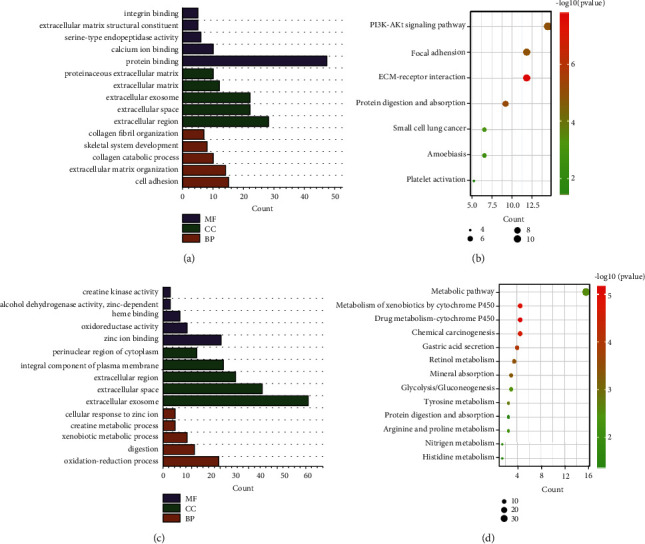
Annotation of differentially expressed genes (DEGs). The screening results were divided into two groups, upregulated genes and downregulated genes, for enrichment analysis by a database for annotation, visualization, and integrated discovery (DAVID). Gene Ontology (GO) terms included biological processes (BP), cellular component (CC), and molecular function (MF), and the top five most significant items in each group and Kyoto Encyclopedia of Genes and Genomes (KEGG) results were illustrated. (a) and (b) The upregulated gene group. (c) and (d) The downregulated gene group.

In order to further understand the function and mechanism of these DEGs, DAVID was employed to analyze GO terms including biological process (BP), cell component (CC), and molecular function (MF), as well as KEGG pathway, and the results were illustrated. In the BP category, the upregulated gene enrichment GO terms were mainly cell adhesion (GO: 0007155) with *p* = 5.09*E* − 09 and extracellular matrix organization (GO: 0030198) with *p* = 1.33*E* − 12, while the downregulated gene enrichment GO terms were mainly oxidation-reduction process (GO: 0055114) with *p* = 5.22*E* − 07, digestion (GO: 0007586) with *p* = 3.21*E* − 12, and xenobiotic metabolic process (GO: 0006805) with *p* = 1.70*E* − 07. Besides, in the CC category, the upregulated gene enrichment GO terms were mainly extracellular region (GO: 0005576) with *p* = 5.56*E* − 11, extracellular space (GO: 0005615) with *p* = 5.09*E* − 08, extracellular exosome (GO: 0070062) with *p* = 0.0032, and extracellular matrix (GO: 0031012) with *p* = 2.91*E* − 08, while the downregulated gene enrichment GO terms were mainly extracellular exosome (GO: 0070062) with *p* = 9.58*E* − 08, extracellular space (GO: 0005615) with *p* = 2.36*E* − 09, extracellular region (GO: 0005576) with *p* = 0.003517504, and integral component of plasma membrane (GO: 0005887) with *p* = 0.015674589. Moreover, in the MF category, the upregulated gene enrichment GO terms were mainly protein binding (GO: 0005515) with *p* = 0.009744135, while the downregulated gene enrichment GO terms were mainly zinc ion binding (GO: 0008270) with *p* = 0.003724062 (Figures 2(a) and 2(c)). As for the KEGG pathway, the most significant results were 31 downregulated genes involved in metabolic pathways (Figures 2(b) and 2(d)).

### 4.3. miRNA-mRNA-TF Regulatory Network Construction

**Figure 3:**
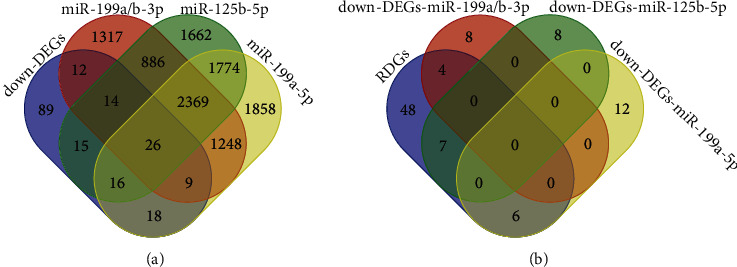
Screening of miRNA-mRNA-TF regulatory network nodes. (a) Among the 199 common downregulated DEGs (down-DEGs) screened from the mRNA expression profile, the overlapping genes with the potential targets of differentially expressed miRNAs (DEMs) were identified. (b) The DEMs targets overlap with the RFX6 downstream genes (RDGs).

**Figure 4:**
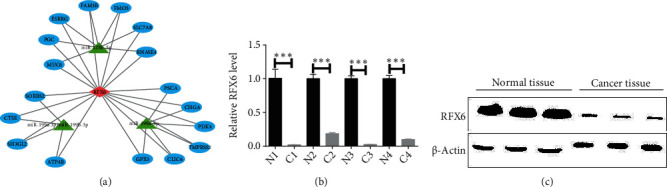
Construction of miRNA-mRNA-TF regulatory network and validation of RFX6 expression level. (a) Three miRNAs (miR-199a-3p/miR-199b-3p, miR-125b-5p, and miR-199a-5p) and transcription factor (TF) RFX6 coregulate gene expression in the miRNA-mRNA-TF regulatory network, and miRNAs negatively regulate their expression levels by binding to the mRNA 3′-UTR region of 17 genes. RFX6 activates transcription by forming a heterodimer with RFX3 and binding to the X-box in the target gene promoter. (b) and (c) Compared with normal gastric tissue, RFX6 was significantly downregulated in GC tissue.

In order to construct miRNA-mRNA-TF regulatory network, mipathDB V2.0 was used to predict the list of potential target genes of miRNAs and compare the list of lower DEGs, and the results showed that the potential genes of miR-199a-3p/miR-199b-3p, miR-125b-5p, and miR-199a-5p were 61, 71, and 69 in the list of downregulated genes, respectively (Figure 3(a)). The results of the CHEA3 prediction show that RFX6 ranks highest among the TFs involved in downregulated DEGs (Table 2). Moreover, in the list of downregulated DEGs, the potential target genes of miR-199a-3p overlap with the RFX6 downstream genes (RDGs) in 4 genes, namely, SH3GL2, ATP4B, CTSE, and SORBS2. The potential target genes of miR-125b-5p overlapped with RDGs in 7 genes, which were SLC7A8, RNASE4, ESRRG, PGC, MUC6, FAM3B, and FMO5. The potential target genes of miR-199a-5p overlapped with RDGs in 6 genes, which were CHGA, PDK4, TMPRSS2, CLIC6, GPX3, and PSCA (Figure 3(b)). Finally, the miRNA-mRNA-TF regulatory network was constructed based on the above screened 17 mRNAs, 3 miRNAs, and 1 TF by Cytoscape 3.8.2 software (Figure 4(a)).

### 4.4. Validation of Significant TF in GC Tissues

In order to further verify the accuracy of the above bioinformatics analysis results, the mRNA and protein levels of the significant regulatory factor RFX6 were verified in 4 pairs of GC tissues and normal tissues by RT-qPCR and western blot. RFX6 activates transcription by forming a heterodimer with RFX3 and binding to the X-box in the target gene promoter, so the expression of RFX6 is positively correlated with downstream genes. The validation results showed that the expression of RFX6 mRNA and protein was significantly downregulated, which suggested that the expression of RFX6 was positively correlated with the expression of downstream genes (Figure 4(b)). Therefore, the above bioinformatics analysis results are proved to be credible from the perspective of experiment.

## 5. Discussion

According to the data released by the Global Cancer Observatory (GCO), in 2020, there were 1,9292,789 new cancer patients in the world, among which 1,089,103 were GC patients, accounting for 5.6%, ranking the fifth. The research on the mechanism of GC and the development of new drugs have always been the shining research direction of medical and scientific researchers.

In this study, DEGs and DEMs were screened out from the existing mRNA and miRNA expression profiles, and the potential targets of DEMs and TF of DEGs were predicted through the database, so as to construct miRNA-mRNA-TF regulatory network and further understand the pathogenesis of GC. First of all, in the 4 mRNA expression profiles containing 210 GC tissues and 118 normal tissues, 275 DEGs were screened using adjust *p* value < 0.05, ∣logFC | >1 as the criterion, among which 76 genes were significantly upregulated in GC tissues compared with normal tissues, while the remaining 199 genes were significantly downregulated in GC tissues compared with normal tissues. Second, to further understand the mechanism of DEGs in biological processes, GO terms and KEGG pathway enrichment analysis were conducted for the upregulated genes and downregulated genes, respectively, and the results showed that the downregulated genes with a higher proportion of DEGs had more obvious enrichment. Then, using adjust *p* value < 0.01, ∣logFC | >1.9 as the criterion, three miRNAs, including miR-199a-3p/miR-199b-3p (adjust *p* value = 3.02*E*-04, log FC = 2.15675), miR-125b-5p (adjust *p* value = 1.58*E*-04, log FC = 1.98925), and miR-199a-5p (adjust *p* value = 9.38*E*-06, log FC = 1.9549), were screened out from a miRNA expression profile containing 20 GC tissues and 20 normal tissues, which were overexpressed in GC tissues relative to normal tissues. After screening the DEMs, to explore the regulatory relationship between miRNA and mRNA and construct miRNA-mRNA regulatory network, the potential targets of miRNAs were predicted by the mipathDB V2.0 data platform, and some of the 199 low-expressed genes that might be regulated by miR-199a-3p/miR-199b-3p, miR-125b-5p, and miR-199a-5p were obtained by using the Venn diagram. Finally, the prediction of TFs, 199 significantly low-expressed genes were used to predict key TFs that played an important role in the progression of GC cases. The prediction results showed that RFX6 ranked the highest in the average of multiple databases, suggesting that RFX6 may play a key regulatory role in the pathogenesis of GC. RT-qPCR results confirmed that the expression of RFX6 was significantly changed in clinical GC tissues.

miRNAs affect gene expression by regulating the translation and degradation of mRNA, and the promotion or inhibition effect of this mechanism on cancer cells has been reported in recent years [33]. It was found by retrieval that the significantly upregulated expression of miR-199a-3p, miR-125-5p, and miR-199a-5p in GC cells and tissues analyzed in this study has been reported many times. In 2003, a computational prediction of miR-199a's identity was made based on the conservatism of miR-199a among humans, mouse, and puffer fish [34]. The expression of miRNA in zebrafish was verified, and the ends of the miRNA were localized by cloning. The two miRNA sequences were named miR-199a and miR-199a∗ (from the 3 ‘arm), respectively. It has been reported that both of these two miRNA expression forms are expressed in humans and are named as miR-199a-5p and miR-199a-3p, respectively [35]. Functional studies have shown that miR-199a-3p can significantly promote the proliferation and inhibit apoptosis of GC cells in vivo and in vitro. It has been found in clinical studies that the overexpression of miR-199a-3p is associated with tumor size, Lauren stage, depth of invasion, lymph node metastasis, distant metastasis, TNM stage, and prognosis [36]. Moreover, in stage I, II, and III tumors, the high expression of miR-199a-3p is associated with a significantly reduced 5-year survival rate. In addition, luciferase report assay demonstrated that miR-199a-3p directly targeted the expression level of ZHX1 regulatory protein. Therefore, researchers hypothesize that the oncogenic activity of miR-199a-3p may be related to the direct targeting and inhibition of ZHX1 [37]. Similar to miR-199a-3p, miR-199a-5p can promote the migration and invasion of GC cells, and its expression level is related to tumor diameter, lymph node metastasis, and TNM stage. However, the difference was that the expression level of miR-199a-5p was not associated with Lauren staging and distant metastasis [38]. Different from miR-199a-3p and miR-199a-5p, the role of miR-125b-5p in the pathogenesis of GC is not fully understood, but the identification results at the cell level and tissue level indicate that miR-125b-5p is significantly upregulated during the development of GC [39]. In addition, in a study on drug resistance, it was found that STAT3 may be a potential target of miR-125b-5p, and the signaling cascades involved in the regulation of chemotherapy drug resistance in GC [40].

In addition to these identified miRNAs, other mRNAs in the miRNA-mRNA-TF regulatory network have also been individually reported, in addition to CTSE, SORBS2, TMPRS, and RNASE4. For example, as a negative regulator of Wnt signaling pathway, ESRRG plays a tumor suppressor role in GC [41]. Moreover, the *β* subunit of the gastric H^+^, K^+^-ATPase encoded by ATPase H^+^/K^+^ Transporting Beta Subunit (ATP4B) are decreased in GC cells and tissues due to the interaction between DNA methylation and histone acetylation [42, 43]. Previous studies have found that if ATP4B expression level is restored, it can inhibit the proliferation, activity, migration, invasion, tumorigenicity, and induction of apoptosis of GC cells, and its tumor-suppressive effect may be played through the regulation of mitochondrial metabolism and apoptosis pathway [44].

Besides, pepsinogen C (PGC) belongs to the aspartic protease family, which is expressed in situ in gastric mucosa, expressed in serum, and expressed ectopic. It is secreted by the gastric host cells and has an activation effect on pepsin C and can digest polypeptides and amino acids [45]. In the process from superficial gastritis to atrophic gastritis and finally to the formation of GC, the positive level of PGC continues to decline, which indicates that the in situ expression of PGC may have a negative correlation with the formation of GC [46]. And the results showed that the expression of MUC6 was decreased in GC tissues, but surprisingly, there was no significant difference between the expression of MUC6 in GC tissues and the gender, tumor site, lymphatic infiltration, clinical stage, metastasis, Lauren intestinal type, diffuse type, and mixed type GC tissues [47].

As for the only TF, RFX6, a member of the RFX (regulatory factor X-box binding) family of winged-helix transcription factors [48], which is downstream of Neurog3 and upstream of many other islet transcription factors in the islet development factor hierarchy, is required for differentiation of four of the five islet cell types [49]. In prostate cancer, on the one hand, clinical data showed that the upregulated expression of RFX6 was associated with the risk of tumor progression, metastasis, and biochemical recurrence [50]. In addition, RFX6 has also been reported to be differentially expressed in melanoma, liver cancer, GC, and other cancer tissues, but the further molecular mechanism remains to be explored.

## 6. Conclusion

In conclusion, in this study, 76 upregulated DEGs, 199 downregulated DEGs, and 3 upregulated DEMs (miR-199a-3p/miR-199b-3p, miR-125b-5p, and miR-199a-5p) were identified from the expression profiles of mRNA (GSE26899, GSE29998, GSE51575, and GSE13911) and 1 miRNA (GSE93415), respectively. Through database prediction and gene list comparison, it was found that among the 199 downregulated DEGs, 61, 71, and 69 genes were the potential targets of miR-199a-3p/miR-199b-3p, miR-125b-5p, and miR-199a-5p, respectively. 199 downregulated DEGs were used as the gene list for the prediction of key TFs, and the results showed that RFX6 ranked the highest. The potential target overlap genes of miR-125b-5p and miR-199a-5p were 4 (SH3GL2, ATP4B, CTSE, and SORBS2), 7 (SLC7A8, RNASE4, ESRRG, PGC, MUC6, Fam3B, and FMO5), and 6 (CHGA, PDK4, TMPRSS2, CLIC6, GPX3, and PSCA), respectively. Finally, we constructed a miRNA-mRNA-TF regulatory network based on the above 17 mRNAs, 3 miRNAs, and 1 TF and verified by RT-qPCR and western blot results that the expression of RFX6 was downregulated in GC tissues. These identified miRNAs, mRNAs, and TF have a certain reference value for further exploration of the regulatory mechanism of GC. It is worth noting that, given the complexity of miRNA and TF crosstalk, these regulatory relationships need to be treated with caution.

## Figures and Tables

**Table 1:** Screening of differentially expressed genes (DEGs).

	Genes
Downregulated	IGF2BP3 COL1A1 CCNE1 FNDC1 S100A3 COL5A2 THY1 MARVELD3 SPP1 OLR1 ITGA2 TIMP1 BGN COL8A1 CDCA5 CKS2 TEAD4 MMP11 CDH3 MMP3 CRABP2 KRT80 FSCN1 LRP8 LIPG BMP1 PLA2G7 MFAP2 TFAP2A COL4A1 TGIF1 SULF1 IL32 LAMC2 RARRES1 CLDN1 IGSF6 HAVCR2 APOC1 GINS2 COL12A1 ESM1 PLAU SERPINB5 TGM2 FOXC1 FZD2 CST1 CLDN7 NDUFA4L2 HOXA10 ETV4 IER5L CAMK2N1 TNFRSF12A ISG15 ANGPT2 TMEM158 IFITM3 SERPINH1 FAP GJB2 THBS2 S100A10 SERPINE1 MSLN LY6E UPP1 COL6A3 COL10A1 F12 COL5A1 PMEPA1 HOXC6 CTHRC1 FJX1

Upregulated	EPB41L4B COL4A5 ISL1 GHR MAOA ZNF385B PRDM16 KIAA1324 GKN1 GHRL CELA3A APOBEC2 SULT1C2 FCGBP GPRC5C KCNJ13 AKR7A3 IRX3 DGKD SORBS2 KLF4 MT1G CYP3A5 GC ALDH6A1 C5 VSIG1 PDGFD FAM3B KIT NTN4 TNFRSF17 CA9 SH3BGRL2 CBS ADH1C NPY IGFBP2 PIK3C2G SLC9A2 GCNT2 SST PLCXD3 ECHDC3 XYLT2 TPD52L1 ZBTB7C GGT6 PDK4 CXCL17 LYPD6B NR3C2 PDIA2 SLC22A23 TCEAL2 MT1E ANXA10 CA4 ADHFE1 ADAM28 MUC6 OSBPL7 KRT20 HRASLS2 CTSE GIF ABCC5 RNASE1 MT1F SOSTDC1 ACACB RORC PNPLA7 VSIG2 FAM46C CHRM3 TMED6 SH3GL2 SCNN1G CKMT2 PSCA KCNJ16 REG3A TFF1 RPRM CYFIP2 FAM3D CKM FBXL13 RNASE4 ST6GALNAC1 DERL3 PTGER3 COBLL1 DNASE1 CXCL14 LDHD PROM2 FBP2 AKR1B10 SOX21 ARL14 AADAC GSTA4 ATP4A POU2AF1 UGT2B15 CKB CD36 LRRC17 RAP1GAP IGFALS TSC22D3 PIGR CDH2 MAL AQP4 CHGA RAB26 MT1M ESRRG APLP1 VILL TMEM171 CPA2 ABCA8 RAB27B FA2H ADH7 PDILT AMPD1 SULT2A1 GAMT TFF2 CCKBR HPGD RBPMS2 ARRDC4 SCGB2A1 SLC7A8 ADA TMPRSS2 DUOX1 LIPF DPT GUCA2B CYP4X1 CAPN13 TCN1 EPN3 SLC2A12 SORBS1 ENPP5 LIFR NQO1 CD79A CYB5R1 CYP2C18 FGA ALDH3A1 FUT9 PTGR1 AZGP1 ALDH1A1 FMO5 IRX2 CA2 LTF GATA5 ATP4B BCAS1 SIDT2 SLC26A9 ADH1A CHIA METTL7A FAM189A2 SSTR1 PGC MYRIP FABP4 KCNE2 FAM107A GPX3 PPP1R3C MT1H SIGLEC11 PTGS1 ALDOB KLK11 DNER MAMDC2 C1orf116 CLDN18 HDC RASSF6 SELENBP1 CLIC6 TRIM50

**Table 2:** The top 10 transcription factors of prediction results.

Rank	Transcription factors	Mean rank
1	RFX6	12
2	FOXA3	14
3	TFCP2L1	15.33
4	GATA5	18.67
5	MYRFL	26
6	ISX	26
7	FOXA2	26.5
8	ISL1	30.67
9	CDX1	31.33
10	FOXA1	34

This prediction is the result obtained by integrating multiple databases. Different databases have different rankings, so the higher the mean rank is, the more accurate it will be.

## Data Availability

The datasets of GSE26899, GSE29998, GSE51575, GSE13911, and GSE93415 were downloaded from the GEO database.
